# Primary Literature in the Undergraduate Immunology Curriculum: Strategies, Challenges, and Opportunities

**DOI:** 10.3389/fimmu.2019.01857

**Published:** 2019-08-07

**Authors:** Jason S. Rawlings

**Affiliations:** Biology Department, Furman University, Greenville, SC, United States

**Keywords:** primary literature, undergraduate, discussion, strategy, opportunity, challenge

## Abstract

Immunology is a rapidly advancing and expanding field that is regularly highlighted in the lay media, whether it be checkpoint blockade immunotherapy winning the Nobel Prize, CAR-T cells in the treatment of cancer, or the latest anti-inflammatory/immunomodulatory medication advertised directly to consumers. Advances such as these not only transform the way we think about immunology, they also illuminate how knowledge of the immune system can be harnessed to impact public health. Immunology is also a vast subject, with thousands of articles published each year that contribute to our understanding of complex processes such as inflammation, pathogen recognition, and self-tolerance, Taken together, these observations pose significant challenges to teaching immunology in the undergraduate classroom. To meet this challenge, instructors can use primary literature as a means to introduce cutting-edge discoveries that have not yet found their way into textbooks, link what students are learning to what they are exposed to in lay media, and ultimately provide added depth to the students' understanding of the immune system all while illustrating how clinical advances are fundamentally dependent on basic research studies. Furthermore, the addition of primary literature to the curriculum can enhance student enthusiasm for learning immunology and can provide an excellent platform for students to gain critical thinking and analytical skills. Presented here are strategies, challenges, and opportunities in the use of primary literature to effectively augment the immunology curriculum in the undergraduate classroom. Topics include selecting papers to read, teaching students how to read scientific literature, and assessing student learning.

## Introduction

Primary literature is an enticing pedagogical tool, as its incorporation into the undergraduate curriculum has been shown to improve scientific literacy (1) and enhance critical thinking skills (2), while providing an excellent platform to teach students how to generate a hypothesis, design experiments, and evaluate data (3). In addition to enhancing learning outcomes, primary literature can also bridge the gap between dated information in textbooks and emerging ideas and concepts. A number of strategies for incorporating primary literature into the science curriculum have been investigated (4–7). The goal of this perspective is to present additional strategies to assist those interested in using a class discussion format to evaluate primary literature as part of the undergraduate immunology curriculum that are aimed to maximize opportunities for student learning and engagement, while minimizing the associated challenges that arise (summarized in Table 1). The strategies presented here can be used in isolation or in conjunction with these other methods.

**Table 1:** Summary of challenges and opportunities associated with incorporating primary literature into the undergraduate curriculum.

	**Challenges**	**Opportunities**
Fitting primary literature into the curriculum	• Requires significant contact hours • Spacing papers out in curriculum • Deciding on learning outcomes	• Highlight emerging concepts • Provide depth beyond the textbook • Great change of pace for instructor and students • Improving scientific literacy
Selecting papers to read	• Gauging student preparedness • Technical difficulty of the paper • Conceptual difficulty of the paper	• Highlight how scientists gain knowledge • Relate papers to current events • Relate papers to student career aspirations
Implementation	• Achieving student buy in • Getting students to evaluate the actual data	• Teaching students about experimental design • Guest speaker to lead discussion • Improve critical thinking and analytical skills

## Fitting Discussion of Primary Literature into the Curriculum

As immunology is a very complex subject, most textbooks contain far more content than what can be covered in a single semester. Thus, the challenge is to figure out how many contact hours to devote to primary literature while still providing a comprehensive immunology curriculum. I have found that the selection of three papers to read throughout the semester provides the best balance between the gains of reading primary literature and the number of valuable contact hours used in the process. In terms of contact hour commitment, you should expect to spend at least 1 contact hour laying down the logistics (described below), your expectations for learning outcomes, and providing instruction on how to read a scientific paper. Additional time will be needed to discuss common techniques and model systems (described more below). The actual discussion of each paper can take roughly 2 h, depending on the papers selected and the depth of the discussion. Thus, to discuss 3 papers throughout the semester, you can plan on a total of ~8 contact hours. Because Immunology at Furman includes a laboratory component, I commit lab sessions as needed without sacrificing too much course content. For reference, Immunology at Furman is comprised of 40 lecture contact hours and 42 lab contact hours; therefore, I commit roughly 10% of total contact hours to primary literature.

## Know Your Students

Before embarking on using primary literature in the immunology curriculum, it is important to have a firm handle on the skills and abilities of your students. At Furman University, Immunology is an upper-level elective course within the Biology major. Students enrolling into the course typically have a strong foundation in genetics and some exposure to cell biology, both gained through three prerequisite courses that serve as the introduction to the major. Students will also know the general anatomy of a scientific paper, but may not have any experience reading a cell/molecular biology paper; therefore, I cannot assume students have a high level of scientific literacy. In terms of interests, the vast majority of students who take Immunology at Furman are on various pre-health career tracks or are interested in biomedical science careers. As such, it is not surprising that students will have the most interest in papers where the translational/clinical link is most apparent. This does not mean that I won't have them read basic science papers, it simply means I will need to help draw the connection between the science and the clinic so they can appreciate the relevance of the paper. This is important, as I have found that if students have “buy in” to what they are reading, they will be more engaged, resulting in a deeper discussion of the paper, and ultimately a deeper understanding of the science.

## Selecting Papers to Read

I have students read one paper from each of the three main thrusts of the Immunology course curriculum: innate immunity, adaptive immunity, and applied immunology. This strategy allows for the papers to be evenly spread out throughout the semester, and also highlights the equal importance of all three areas of immunology. Importantly, this strategy also provides the most flexibility for the instructor in planning out the curriculum and provides a great change of pace for both the instructor and the students.

Great care must be taken in selection of papers to read. The ideal paper will be able to stand alone, in that it has sufficient background in the introduction such that the students can reasonably connect the science to what has been covered in class (or I will adjust coverage in class to make the connection more apparent). The paper will have the right balance between technical difficulty and the complexity of the science itself. Much of this decision will rest on the strengths of the students (see above). I will avoid using papers that are incredibly “data heavy” or have methods that are not clearly spelled out, as undergraduates may have difficulty digesting the data. Ideally, the paper will utilize multiple modalities or approaches to address a clearly articulated hypothesis. I try to select a set of papers that collectively utilize a broad array of techniques so that students are exposed to a variety of data types. When possible, I like to select papers that have timely relevance. For example, choosing a checkpoint blockade paper will not only highlight the recent award of the Nobel Prize to James Allison and Tasuko Honjo, it will allow students to identify with the direct application of their findings in discussing the recently approved therapeutics based on their discovery. Additionally, such a paper illustrates how clinical advances are absolutely dependent on basic science studies.

The greatest care must be taken in selecting the first paper students will read, as this will set the tone for the remainder of the semester. This selection can be challenging, as students will not have had much exposure to immunology at this point in the semester and even more so if the students are relative novices at reading and discussing primary literature. I like to choose a paper that is visually appealing and has data that is relatively easy to digest. A great example is to use a paper on neutrophil extracellular traps (NETs). In my experience, students have found the idea that a cell will extrude its DNA to trap microbes to be intriguing. Because much of the data is visual in nature, students will be able to not only digest it easily, they will be fascinated by it. Importantly, I cover an overview of cells of the immune system very early in the course, thus students can begin reading the paper almost immediately.

## Implementation

Early in the semester, I recommend devoting 1–2 contact hours to covering the basic anatomy of a scientific paper (if needed), common techniques (e.g., flow cytometry, transgenics) and perhaps introduce model systems that are utilized in the selected papers. In addition, this time should be used to lay out overall expectations, including how learning will be assessed. It is important to emphasize to the students that it will take multiple in-depth readings of the paper to understand the science presented. I devote one of the first lab sessions of the semester to these tasks.

Foundational material will need to be covered to prepare students for reading primary literature (e.g., discussing basic functions of neutrophils before assigning a paper on NETs). The instructor need not alter their pedagogy in the delivery of this content. Once foundational material is covered, I will prompt students to start reading the paper and assign them to write a two-page (double spaced) summary of what they read, giving them 2 weeks to complete the assignment. In the assignment, students are instructed to spend approximately half of one page on the introduction, half of a page on the discussion, and the remainder on the results. I do not have students commit a specific section to the methods. Instead, students are to incorporate methods used as they describe the data. For example: “*When T cells were stimulated with IL-2, expression of GeneX increased significantly, as measured by qRT-PCR.”* When laying out expectations for the paper summary, I am transparent with the students about the fact that it will be extremely difficult to summarize the paper in just two pages; the true goal of the assignment is to force students to write concisely and most importantly, attempt to synthesize what they are reading. I also make it clear to the students that simply embellishing the abstract will not meet my expectations for their summary (see section Assessment of Learning below and Table 2). Furthermore, expectations regarding plagiarism (which students will have already had exposure in previous courses) and ethical behavior regarding the assignment are reinforced.

**Table 2:** Example questions that can be asked to facilitate discussion of primary literature.

**Section**	**Example questions to facilitate discussion**
Abstract/Introduction	• In your own words, can you give a one sentence “elevator pitch” for the main findings of the paper? • What is the main objective of the paper? • What background information would someone need to understand the results of the paper? • What is the overall objective of the study?
Methods/Results	• What biological question is the experiment presented in the figure trying to address? • What are the positive/negative controls? • What are the technical controls? (e.g., loading controls for a western) • What additional controls (if any) are included? • Are any controls missing? • Can you identify any confounding variables? • How does the assay used to generate the data actually measure the phenomenon? • Does the data presented in the figure follow the authors' interpretation? • Is there an alternative/additional experiment you could do to address the same biological question?
Discussion/Critique of paper	• Did the data presented in the paper address the authors' main objective? • Did the authors place their findings into broader context? • What are the main strengths of the paper? • What are the main weaknesses of the approaches used? • What additional experiments could you do to validate the data shown? • If you were to continue this line research, what experiment would you do next?

Concomitant with assigning the paper summary, I open an online discussion forum for students to post questions about the paper. Students are encouraged to ask both technical questions, as well as questions about the biology. Students are also encouraged to answer each other's questions and engage in discussion about what they are reading. I closely monitor the forum, intervening only when student questions go unanswered or if student replies/discussion gets off track. If the forum lacks activity, I will post questions for students to answer to stimulate discussion. Ideally, students will spend ~1 week discussing issues on the online forum and use the second week to complete their summary.

In addition to the online forum, I will make every attempt to directly connect course content with what they are reading as we progress through the curriculum. For example, if I assign a paper that utilizes an OT-I transgenic TCR model, I will talk about how expression of the transgene early in thymocyte development results in virtually all T cells expressing the transgenic TCR when I cover TCR rearrangements and control of receptor expression in class. I will also refer to the OT-I model as a means to test signal strength when we discuss thymocyte selection. The goal is to organically interweave the technical aspects of the paper into what they are learning as a way to show students how immunological concepts can be applied.

The culminating event is the in-class discussion of the paper, for which I will commit at least 2 contact hours. Students are encouraged to bring a copy of their paper summaries to the discussion. The discussion begins by covering the key points in the introduction, making sure that students understand the necessary background information as well as the main objective of the paper. If the paper relies heavily on an advanced technique, a significant amount of time will be spent making sure all students understand how it works, typically expanding on the threads from the online forum. The majority of the discussion will revolve around the results. For each experiment, I will have the students indicate the specific question that is being addressed (and why). Students will then be asked to identify the positive and negative controls and to assess if they are good controls to use for the experiment. Most importantly, I will ask the students to indicate whether the data presented in the figure support the authors' hypothesis. This is critical, as I have found that students will often take the authors' conclusions as absolute truth and typically will not critically evaluate the figures when they write their paper summaries. Importantly, this question forces students to actually think about the data and what it means. Finally, we spend time on the discussion section of the paper, with the goal of putting the findings into broader context. In addition to covering the content the authors' provide, I ask students to identify additional experiments that could be done to support the authors' conclusions, including asking how they might design such an experiment. We also spend time critiquing the science within the paper, as I want to instill in the students that scientists should be critical in evaluating science, even what is presented in a peer-reviewed publication. Examples of questions to ask that may assist in promoting discussion are provided in Table 3.

**Table 3:** Checklist that can be used to develop paper-specific rubrics for grading paper summaries.

**Section**	**Element**
Introduction	Background elements described sufficiently to understand the remainder of the summary The relevance of the study/authors' motivation clearly stated Model systems employed are adequately described The objective of the paper is clearly stated
Methods/Results	All experiments (except supplemental data) are described Technique/method used to obtain data for each experiment is mentioned Attempt made at synthesizing information presented in figures
Discussion	How did the authors place the findings into the context of published literature? How do the authors reconcile any differences with published literature (if applicable)? Did the authors adequately address the objective? What weaknesses (if any) does the paper have? Overall critique of the paper
Formatting/Other	Did summary conform to length limit? Is the summary organized logically? Is the writing concise and clear? Check for plagiarism (including from paper abstract)

For an incredibly enriching experience, consider bringing in the first author or corresponding author of one of the papers to lead the in-class discussion. In the past, I have invited scientists in the context of a traditional campus visit, where I've had the speaker give a typical “research talk” open to the public in addition to leading the discussion of one of their papers in my Immunology class. Each time I've done this, the students really enjoyed being able to ask more deliberate questions (e.g., “why did you think to do that particular experiment?” or “how did you first generate the hypothesis?”). The guest speaker can also give the students insight into the process of doing science that isn't easily gleaned from reading the paper (e.g., pitfalls encountered and how they were overcome). A few words of caution are in order. First, you should let the speaker know that they will most likely not be able to discuss the entire paper in the context of an hour-long session (the remainder of the paper can be discussed at a future class meeting). Second, be prepared that the discussion may very well turn toward topics such as the speaker's career path. Personally, I welcome these turns, and plan accordingly by anticipating using more total time for the discussion. Finally, I give extra preparation for the students (via taking a more active role in guiding discussion on the online forum prior to the speaker's visit) to ensure that the students will make a good impression on the speaker.

## Assessment of Learning

Students are formally assessed via three mechanisms: the paper summary, participation in discussion, and on exams. The paper summary serves as the mechanism to ensure that students have deeply read the paper prior to the in-class discussion. When grading their summary of the introduction, I look to see if the student provides enough background to understand the premise of the paper and that they clearly articulate the overall objective. For the results section, I look for completeness: did the student summarize all of the salient experiments in the paper? Did they identify the technique(s) used to obtain the data? I also gauge whether the student was able to synthesize the information they read. When grading the discussion portion, I look to see if the student can place the findings into a broader context, and whether they understood the major points the authors raise. Importantly, throughout the summary, I do not penalize the students for incorrectly interpreting the data or the authors' conclusions, as it is inevitable that some of the concepts or technical aspects may be too difficult for the student to get on their own reading. Table 2 provides a generalized rubric for grading paper summaries that is augmented with additional elements specific to the particular paper assigned.

Students have the opportunity to participate in the discussion of the paper via two modalities, the online forum and the in-class discussion. I will moderate the online discussion by providing hints or clues to point the discussion in the right direction when questions go unanswered or if student responses are incorrect. If overall activity is low or if there is a particular technical aspect or biological concept that I anticipate students might struggle with, I will proactively post discussion questions that will aid in student understanding. The in-class discussion follows the same philosophy as the online forum, except the goal is to ensure that students gain a thorough understanding of the paper. We will go through every panel of every figure in the paper (typically not supplemental data). For each figure, I make sure students understand the experimental design and are charged with explaining why the data presented in the figure supports the authors' written conclusions in the text. In terms of grading student participation, I value online participation just as much as participation in the in-class discussion. That said, it is my expectation that every student will engage in the in-class discussion. For both the online forum and in-class discussion, I am interested in seeing thought-provoking questions and well-thought out answers. I am clear with students from the outset that I am interested in quality not quantity. Students can earn up to 30 points throughout the semester for participation, representing 25% of the total points committed to primary literature for the course. Students gain points for either online or in-class participation. During the in-class discussion, I specifically do not allow students who have already maxed out their participation score to respond to questions raised until the rest of the class has had an opportunity to respond. I provide positive reinforcement to all student responses to encourage students to continue participating.

I assess student learning by incorporating questions from the paper on exams. I will typically take a figure from the paper and ask students to evaluate the data, including experimental design. Depending on the paper, I might ask the students follow-up questions about further experiments they might propose. I will also allow students to use a “clean” copy (not marked in any way) of the paper during the exam. In this case, I might ask students to identify the experiment(s) in the paper that address a specific hypothesis.

In terms of points toward the final grade in the course, I make the paper summaries worth 30 points each and students can get up to 10 points for participation in discussions of each paper (online and in-class). If discussing three papers over the course of the semester, collectively this amounts to a bit more than a single exam. I have found this weighting provides sufficient incentive for students to perform at a level needed for a good discussion of the paper and achievement of learning outcomes.

## Concluding Remarks

Successfully incorporating primary literature into the undergraduate Immunology curriculum presents unique challenges and opportunities for the Instructor. Perhaps the most important challenge is to clearly identify the learning goals you wish to achieve. Once the goals are established, great care must be taken to select papers that will meet those goals, are accessible to your students, and will fit into the curriculum. Importantly, implementation must include mechanisms that encourage students to read the literature with the depth necessary to understand the science and provide means to clarify some of the technical and conceptual issues associated with the paper prior to the in-class discussion. If all of the above is done well (a tall task!), you can expect that your students will have learning gains in addition to learning immunology beyond the textbook to include enhanced analytical and critical thinking skills, improved scientific literacy, a greater appreciation for how scientific knowledge is obtained, and greater enthusiasm for learning other aspects of immunology. In my experience, most students can see the immediate benefit of reading and evaluating primary literature as evidenced by unprompted comments such as these on end-of-course evaluations:

“The journals were one of my favorite parts of the course”

“I loved the papers we had to discuss.”

“…the paper discussions were very helpful and will definitely help us in the future as we begin to conduct our own research and review published work in our future careers.”

“the papers were well chosen and extremely interesting.”

“[Suggestions for improving the course include] additional papers to read”

In addition to all of the above, and perhaps most importantly, the ability to read and understand primary literature will serve your students well beyond the classroom as they will have gained the toolset needed to serve as competent ambassadors of science.

## Author Contributions

The author confirms being the sole contributor of this work and has approved it for publication.

### Conflict of Interest Statement

The author declares that the research was conducted in the absence of any commercial or financial relationships that could be construed as a potential conflict of interest.
